# Assessment of the Mutagenic Activity of Extracts of Brazilian Propolis in Topical Pharmaceutical Formulations on Mammalian Cells *In Vitro* and *In Vivo*

**DOI:** 10.1093/ecam/nen049

**Published:** 2011-04-14

**Authors:** Juliana Marques Senedese, Aline Rafaela Rodrigues, Michelle Andrade Furtado, Viviane Dias Faustino, Andresa A. Berretta, Juliana M. Marchetti, Denise Crispim Tavares

**Affiliations:** ^1^Universidade de Franca, Av. Dr Armando Salles de Oliveira, 201, 14404-600 Franca, São Paulo, Brazil; ^2^Faculdade de Ciências Farmacêuticas de Ribeirão Preto, Universidade de São Paulo, Ribeirão Preto, São Paulo, Brazil; ^3^Apis Flora Industrial e Comercial Ltda, Ribeirão Preto, São Paulo, Brazil

## Abstract

Propolis possesses various biological activities such as antibacterial, antifungal, anti-inflammatory, anesthetic and antioxidant properties. A topically applied product based on Brazilian green propolis was developed for the treatment of burns. For such substance to be used more safely in future clinical applications, the present study evaluated the mutagenic potential of topical formulations supplemented with green propolis extract (1.2, 2.4 and 3.6%) based on the analysis of chromosomal aberrations and of micronuclei. In the *in vitro* studies, 3-h pulse (G_1_ phase of the cell cycle) and continuous (20 h) treatments were performed. In the *in vivo* assessment, the animals were injured on the back and then submitted to acute (24 h), subacute (7 days) and subchronic (30 days) treatments consisting of daily dermal applications of gels containing different concentrations of propolis. Similar frequencies of chromosomal aberrations were observed for cultures submitted to 3-h pulse and continuous treatment with gels containing different propolis concentrations and cultures not submitted to any treatment. However, in the continuous treatment cultures treated with the 3.6% propolis gel presented significantly lower mitotic indices than the negative control. No statistically significant differences in the frequencies of micronuclei were observed between animals treated with gels containing different concentrations of propolis and the negative control for the three treatment times. Under the present conditions, topical formulations containing different concentrations of green propolis used for the treatment of burns showed no mutagenic effect in either test system, but 3.6% propolis gel was found to be cytotoxic in the *in vitro* test.

## 1. Introduction

Injuries caused by burns are the third most frequent cause of accidental death in all age groups, with 75% of these lesions resulting from the victim's action and occurring at home. In the United States, 70 000 individuals are hospitalized every year with severe injuries caused by thermal trauma [1]. Burns are caused by physical (temperature, radiation and electricity) and chemical agents (acids and alkalis), and vary in degree according to the intensity or concentration of the causal agents and extent of exposure [2].

The skin is a biological interface between the environment and organism and represents the first line of defense against external noxious stimuli such as ultraviolet light, visible irradiation, pro-oxidant chemicals, infection and ionizing radiation [3]. Topical administration of antioxidants provides an efficient way to improve the endogenous cutaneous protection system [4].

A topically applied product based on propolis was developed using as vehicle a polymeric system consisting of hydrophilic poly(oxyethylene)-poly(oxypropylene)-poly(oxyethylene) polymers (Poloxamer 407), inert and atoxic substances able to generate thermoresistant gel-like colloidal solutions in the presence of water, with the latter affecting the behavior of the solution and the molecular diffusion of the active substance [5, 6]. The low toxicity and reduced skin irritation of Poloxamer 407 encouraged the evaluation of potential dermatological applications of these gels, particularly in the treatment of burns [7], in view of their advantages such as easy application and removal of the preparation, as well as the possibility of maintaining the therapeutic concentration at the site of application. A surgically induced injury was found to be completely healed within a period of 7 days. In addition, gel containing 3.6% propolis led to evident epithelial reconstruction after 3 days, with the observation of organized connective tissue fibers and numerous fibroblasts [8].

Propolis is produced by bees from plant resins and exudates, and its colour, consistency and chemical composition are intimately related to the flora visited by the bees and to the season during which it is collected [9]. At present, more than 300 compounds, mainly polyphenols, have been identified as constituents of propolis [10]. Most polyphenols are flavonoids, followed by phenolic acids, esters, aldehydes, ketones, and so forth. [11]. Propolis possesses various biological activities such as anti-inflammatory [12], antibacterial [13], antifungal [14], anesthetic [15] and antioxidant properties [16, 17]. In addition, it has been used in topical applications as a tissue regenerating agent, which is one of its most popular uses in the world today [18]. However, propolis contains some compounds which are toxic and induce hypersensitivity reactions. The main target organ is the skin, with contact dermatitis being a common manifestation [19].

The mechanisms responsible for the improvement of health conditions observed with the use of propolis in folk medicine are still unknown. To guarantee the safe application of propolis in the pharmaceutical industry, it is important to determine whether topical formulations supplemented with green propolis extract used for the treatment of burns induce DNA damage. Therefore, the aim of the present study was to assess the possible mutagenic effect of these formulations by *in vitro* analysis of chromosomal aberrations in Chinese hamster ovary (CHO) cells and by the *in vivo* micronucleus test in Wistar rats.

## 2. Materials and Methods

### 2.1. Preparation of Test Formulations Containing Propolis Extract

The topical formulations containing standard green propolis extract (SPE-AF) used for the treatment of burns were provided by Apis Flora Comercial e Industrial Ltda., Ribeirão Preto, São Paulo State, Brazil (Patent number PI 0405483-0, published in Revista de Propriedade Industrial no 1778 from January 02, 2005). Green propolis extract was prepared from propolis in natura produced in the region of Oliveira (State of Minas Gerais, Brazil), a region rich in native *Baccharis dracunculifolia*. The gels were prepared on a weight basis using the cold method according to Schmolka (1972). Concentrations of Poloxamer 407 and of SPE-AF are expressed as percent weight (w/v). An appropriate amount of Poloxamer 407 was slowly added to cold distilled water (5°C) under constant stirring. The polymer dispersion was kept in the refrigerator until a clear solution had been formed (6–12 h). Appropriate amounts of SPE-AF and polyoxyl castor oil were prepared to yield 1.2, 2.4 and 3.6% (w/v) of dry propolis extract and then dissolved in the cold solution. Two control samples were prepared, one consisting of the polymer dispersion and the other of the solubilizing agent used to obtain a clean gel.

### 2.2. Analysis of Propolis Extract by HPLC

The chromatographic analysis of green propolis extract was performed using a High Performance Liquid Chromatograph (HPLC) Shimadzu equipped with controller SCL-10A*vp*, three pumps LC-10AD, detector diode-array model SPD-M10A*vp* and software controller Shimadzu Class-VP version 5.02. A Shim-Pack CLC-ODS (M), Shimadzu column (4.6 mm × 250 mm, particle diameter of 5 *μ*m, pore diameter of 100 Å) was used. The mobile phase consisted of a buffer solution in pump A (93.9% water, 0.8% acetic acid, 0.3% ammonium acetate, 5% methanol) and acetonitrile in pump B. The elution was undertaken using a linear gradient of 25–100% of B in 60 min at a flow-rate of 1.0 mL min^−1^. Detection was performed at 280 nm.

The phenolic compounds were identified by comparison with the authentic chromatographic standards available at the compounds library of the Pharmacognosy Laboratory of the School of Pharmacy of Ribeirão Preto, São Paulo, Brazil, comparing UV spectra and considering both the maximum lambda and the relative area obtained with the use of two wavelengths (A_280/320_).

The crude propolis extract was dissolved in methanol (HPLC grade) to obtain a concentration of 1 mg mL^−1^. Before analysis, all samples were centrifuged at 1300 rpm and filtered through a 45-*μ*m filter.

### 2.3. Chromosomal Aberrations Assay in CHO Cells

CHO_9_ cells were kindly supplied by the Laboratory of Cytogenetics and Mutagenesis, University of São Paulo, Ribeirão Preto, São Paulo, Brazil. Cells were maintained as monolayers in plastic culture flasks (25 cm^2^) in HAM-F10 (Sigma-Aldrich, St. Louis, MO, USA) and D-MEM (Sigma-Aldrich) (1 : 1) culture media supplemented with 10% fetal bovine serum (Nutricell), antibiotics (0.01 mg mL^−1^ streptomycin and 0.005 mg mL^−1^ penicillin; Sigma-Aldrich), and 2.38 mg mL^−1^ HEPES (Sigma-Aldrich), at 37°C in a BOD type chamber.

Exponentially growing CHO cells were seeded (1 × 10^6^ cells per flask) and allowed to grow for 20 h (approximately 1.5 times the normal cell cycle) [20]. The cultures were treated with 5 mg mL^−1^ of each gel containing different concentrations of propolis (1.2, 2.4 and 3.6%), with this being the concentration limit specified by guidelines for cases in which the molecular weight is unknown or mixtures are being tested. Two treatment protocols were used: 3-h pulse treatment and continuous (20 h) treatment. After the 3-h pulse treatment, the cells were washed twice in phosphate-buffered saline, fresh medium was added and the cultures were incubated at 37°C for an additional 17 h. In continuous treatment, CHO cells were seeded and treated until harvest. The cells were fixed 20 h after the beginning of treatment in both protocols. Doxorubicin (DXR, Pharmacia Brasil Ltda., São Paulo, Brazil) was added to the cultures at concentrations of 1.0 and 2.0 *μ*g mL^−1^ for the continuous and 3-h pulse treatment, respectively, as positive control. Three independent replicates were carried out for each treatment.

Colcemid (Demecolcine, 0.1 *μ*g mL^−1^; Sigma-Aldrich) was added to the culture medium 2 h before fixation. At harvest, the cells were trypsinized (0.025%) and then hypotonized in 1% sodium citrate solution at 37°C for 30 min. The cells were fixed in methanol/acetic acid (3 : 1) and the slides were stained with 5% Giemsa for 5 min.

For the determination of chromosomal aberrations, 100 metaphases were analyzed per culture, for a total of 300 cells per treatment and control, and the aberrations were classified according to Savage [21]. The mitotic index (MI) corresponds to the number of metaphase cells among 2000 cells analysed per culture and is reported as percentage. The MI is expressed as the mean of three replicates. The data obtained were analysed statistically by ANOVA for repeated measures, followed by the Tukey test, with the level of significance set at *α* = 0.05. Gaps were recorded but not included in the statistical analysis since their cytogenetic significance has not been well established.

### 2.4. Micronucleus Assay in Wistar Rats

For the experiments, 30 male Wistar rats (*Rattus norvegicus*, Berkenout, 1769) with an initial body weight of 45 g, obtained from the Central Animal House, Faculty of Medicine of Ribeirão Preto, University of São Paulo, Brazil, were allocated to three treatment times: acute (24 h), subacute (7 days) and subchronic (30 days). The study protocol was approved by the Ethics Committee for Animal Care of the University of Franca (process 121/05).

Since the topical formulation used in the present study is aimed at the treatment of burn injuries, a lesion was created with a punch on the back of animals previously anesthetized by intraperitoneal administration of ketamine, midazolam and acepram [22].

The concentrations of the propolis extract added to the topical formulations used in the present study, as well as the treatment protocol, were established based on previous histological studies regarding the healing effect of the gel [8]. The animals were treated with gels containing the following concentrations of propolis: 1.2, 2.4 and 3.6% w/v. In addition, a group of animals treated with propolis-free gel, a negative control group and a positive control group (50 mg cyclophosphamide kg^−1^ body weight) were included. Each treatment group consisted of five animals. These groups were submitted to acute, subacute and subchronic treatments with gels containing propolis or not, with the animals being treated and weighed daily.

The frequency of micronuclei was determined in peripheral blood of Wistar rats according to the technique of MacGregor et al. [23]. Peripheral blood smears were obtained 24 h and 7 and 30 days after the beginning of application of the gels to the dorsal lesions of the animals. The frequency of micronucleated polychromatic erythrocytes (MNPCEs) was determined based on the analysis of 2000 anucleated polychromatic erythrocytes (PCE) per animal. A total of 400 erythrocytes per animal were scored to determine the nuclear division index (NDI, PCE/PCE + NCE [normochromatic erythrocytes]).

Differences in the frequencies of MNPCEs and NDI between groups treated with the different propolis gels at the three exposure times were analysed statistically by the Tukey test, with the level of significance set at *α* = 0.05.

## 3. Results

### 3.1. Analysis of Propolis Extract by HPLC

**Figure 1:**
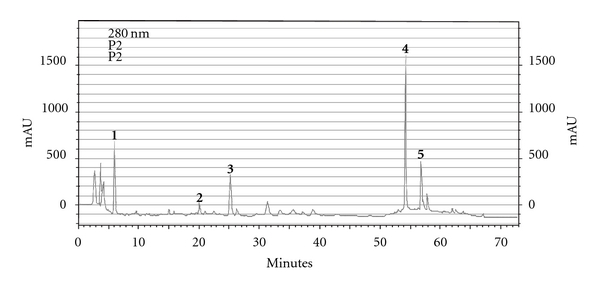
HPLC chromatographic profile of green propolis extract. (**1**) *p*-coumaric acid; (**2**) aromadendrin-4′-methyl ether; (**3)** 3-prenyl-*p*-coumaric acid (drupanin); (**4**) 3,5-diprenyl-*p*-coumaric acid (artepillin C) and (**5**) baccharin.

HPLC analysis of green propolis extract permitted the identification of the following compounds: (i) *p*-coumaric acid; (ii) aromadendrin-4′-methyl ether; (iii) 3-prenyl-*p*-coumaric acid (drupanin); (iv) 3,5-diprenyl-*p*-coumaric acid (artepillin C) and (v) baccharin (Figure 1).

### 3.2. Chromosomal Aberrations Assay in CHO Cells

The results obtained for the 3-h pulse and continuous treatments using gels with different concentrations of propolis and their respective controls are shown in Table 1. Cultures submitted to 3-h pulse treatment with gels containing 1.2 and 2.4% propolis showed a small increase in the number of chromosomal aberrations and altered metaphases compared to the control group, but these differences were not statistically significant. In the continuous treatment, gels containing 2.4 and 3.6% propolis presented slightly higher frequencies of chromosomal aberrations and altered metaphases than the negative control but the difference was not significant (*P* > .05).

No significant differences in the MI were observed between cultures submitted to 3-h pulse treatment with gels containing different propolis concentrations and their respective controls. In the continuous treatment, lower MI were observed for cultures treated with gels containing 2.4 and 3.6% propolis when compared to control, but this decrease was only significant (*P* > .05) for the 3.6% propolis gel (Table 1).

### 3.3. Micronucleus Assay in Wistar Rats

Table 2 shows the mean initial body weight, final body weight and body weight gain during the experimental period. No statistically significant differences in these variables were observed between groups (*P* > .05).

The frequencies of MNPCEs in peripheral blood of animals submitted to acute, subacute and subchronic treatments with gels containing different propolis concentrations are shown in Table 3. Animals submitted to acute treatment with 1.2% propolis gel showed a lower frequency of MNPCEs compared to the other groups, but this difference was not statistically significant. In the subacute treatment, no difference in the frequency of MNPCEs was observed between the groups receiving propolis gels and the negative control. In the subchronic treatment, comparison of the frequency between the negative control and the other groups showed a lower frequency of MNPCEs in the group receiving propolis-free gel and the group treated with 3.6% propolis gel. However, these differences were not significant (*P* > .05). Thus, acute, subacute or subchronic treatment did not result in an increase in the frequency of MNPCEs in animals treated with gels containing different propolis concentrations when compared to the negative control or to animals treated with propolis-free gel.

Comparison of the frequencies of MNPCEs between the different exposure times revealed a nonsignificant reduction in all treatment groups at 7 and 30 days compared to the 24-h treatment. This decrease is probably related to the adaptation of the animal to the housing conditions.

Analysis of the NDI obtained for the acute, subacute and subchronic treatments showed no significant difference in the ratio of polychromatic erythrocytes to total erythrocytes between animals treated with gels for burns containing different propolis concentrations and controls.

## 4. Discussion

From the biological activities found for propolis, the antioxidant activity deserves special interest since it suggests propolis could be successfully applied topically to prevent and treat skin damages. Recently, propolis extract added to topical formulations has been shown to maintain its antioxidant activity, protecting skin against damage caused by free radicals [16].

The antioxidant activity of green propolis has been investigated by Simões et al. [24], who studied the biological effects of different extracts and fractions of green propolis. A correlation was observed between the antioxidant activity and chemical composition of its different fractions, with special emphasis on the presence of flavonoids and *p*-coumaric acid derivatives. The authors concluded that the components of propolis act through different mechanisms sequestering reactive oxygen species. Artepillin C (3,4-diprenyl-*p*-coumaric acid), a major constituent of green propolis, is also an excellent scavenger of free radicals similar to catechins [25].

Tavares et al. [26] studied the mutagenic and antimutagenic effects of the green propolis on CHO cells. The authors showed that, on the one hand, the highest propolis concentration tested resulted in a small but significant increase in the frequency of chromosomal aberrations whereas, on the other hand, the lowest concentration tested significantly reduced the chromosome damage induced by the chemotherapeutic agent DXR. These results indicate that green propolis possesses the characteristics of a “Janus” substance, that is, propolis is mutagenic at higher concentrations, while at lower concentrations it exerts a chemopreventive effect on DXR-induced mutagenicity. Ozkul et al. [27] also reported mutagenic effect of propolis when tested at high concentrations in human lymphocytes.

In the present study, the topical formulations supplemented with green propolis extract for the treatment of burns were assessed *in vitro* for their mutagenic effect on CHO cells and *in vivo* for their capacity to induce micronuclei in peripheral blood. The results obtained in the *in vitro* assay showed that 3-h exposure to these topical formulations did not produce any significant increase in chromosomal aberrations. According to Galloway et al. [20], in the case of a negative result in the 3-h pulse treatment, continuous treatment should be performed. Thus, we submitted CHO cells to continuous treatment after obtaining a negative result in the 3-h pulse treatment. Similarly, 20-h treatment with propolis gels did not result in an increase of chromosomal aberrations compared to the control culture.

Regarding the test system used in the present study, it should be emphasized that the chromosomal aberrations assay in mammalian cell cultures is one of the most widely used methods for the assessment of mutagenic and/or carcinogenic agents [28]. The sensitivity of the test system was demonstrated by the observation of a significant increase in chromosomal aberrations produced by the positive control substance (DXR) and by the fact that negative control values were within the range reported for the CHO *in vitro* test system.

Analysis of the MI showed that gels containing different concentrations of propolis presented no cytotoxic effect, except for the 3.6% propolis gel which was cytotoxic in the continuous treatment. A nonsignificant increase in the number of chromosomal aberrations was also observed in this treatment. According to Galloway et al. [20], an increased osmolarity of the culture medium may cause an increase in the number of chromosomal aberrations. Thus, the increased frequency of chromosomal aberrations observed might be related to the cytotoxicity of gel containing 3.6% propolis. This cytotoxicity might be explained in part by the presence of artepillin C, the most abundant compound identified (Figure 1), which has shown *in vitro* cytotoxic activity in some cell lines. The observed cytotoxicity seemed to be partly attributable to the induction of apoptosis-like DNA fragmentation [29].

It is known that many compounds can yield negative *in vitro* results and positive *in vivo* results because of their indirect action and consequent need for metabolic activation. Furthermore, the possibility that many of these positive results may not be relevant in terms of human exposure [30] should be taken into account. For this reason, in addition to the *in vitro* test, the topical formulations supplemented with green propolis extract for the treatment of burns were also tested for their capacity to induce micronuclei *in vivo* in rat peripheral blood. The results obtained with the *in vivo* test system showed that these gels did not increase the frequency of MNPCEs in peripheral blood of rats submitted to acute, subacute or subchronic treatment. Some considerations regarding the test system used in the present study are important. The micronucleus test is the most widely used *in vivo* assay for the identification of clastogenic and aneugenic agents, and is conducted using the bone marrow or peripheral blood of rodents [31]. In this study the rat peripheral blood was employed because previous histological studies regarding the healing effect of the gels containing different concentrations of green propolis were performed using this species [8].

According to Abramsson-Zetterberg et al. [32], since rats have been used as an animal model in conventional toxicological studies, parallel application of the micronucleus test may be advantageous as an indication of the genotoxic effect in this species. In the case of prolonged exposure of rats, a species commonly used in toxicological tests, various peripheral blood samples for the micronucleus test can be obtained from the same animal. Analysis of micronucleated cells in peripheral blood samples obtained at various times along the experiment provides important supplementary information regarding the time that has elapsed since the induction of micronuclei.

With respect to the route of administration used in the present study, it is important to emphasize that MNPCE analysis is adequate for the assessment of the possible mutagenicity of gels containing different concentrations of green propolis and applied dermally. Itoh et al. [33] used the same test system for the evaluation of the antimicrobial agent quinolone applied dermally to mice. The results showed that the method was a useful tool for the detection of *in vivo* chromosome breaks and for the investigation of the photochemical carcinogenesis of chemicals. Vijayalaxmi et al. [34] observed that jet fuels did not have the potential to induce genotoxicity based on micronucleus studies in the peripheral blood and bone marrow of mice treated dermally.

The increased frequency of MNPCEs observed in animals treated with the known clastogenic agent cyclophosphamide, used as positive control in the present study, indicates that this test system should reveal an increase in the frequencies of MNPCEs in animals treated with gels containing different concentrations of green propolis if the latter were mutagenic. The absence of mutagenicity in rat peripheral blood erythrocytes suggests that these gels are not mutagenic or they are not absorbed systemically when applied dermally.

In the present study, the *in vivo* micronucleus assay confirmed that the topical formulations supplemented with green propolis extract have no mutagenic effect as demonstrated in the *in vitro* test.

In conclusion, under the present conditions topical formulations supplemented with green propolis extract used for the treatment of burns showed no mutagenic effect in either test system, but 3.6% propolis gel was cytotoxic in the *in vitro* test. The present results contribute to a better understanding of the action of propolis on the human organism, and consequently permit the safer use of topical formulations supplemented with green propolis extract in future clinical applications.

## Figures and Tables

**Table 1:** Number of abnormal cells and mitotic index (MI) obtained for CHO cells submitted to 3-h pulse or continuous (20 h) treatment with gels containing different concentrations of propolis and their respective controls.

Treatments	MI (%) ± SD^c^		Abnormal cells ± SD^c^		Aberration frequency	
	3-h pulse	20 h	3-h pulse	20 h	3-h pulse	20 h
Control	6.05 ± 2.00	6.08 ± 1.32	2.00 ± 2.00	3.00 ± 2.00	0.02	0.03
DMSO^a^	4.92 ± 0.40	4.72 ± 2.83	2.00 ± 1.00	3.70 ± 2.34	0.02	0.04
Without propolis	4.26 ± 0.60	5.15 ± 0.02	2.40 ± 1.53	2.70 ± 0.58	0.03	0.03
1.2% Propolis	9.08 ± 0.60	6.77 ± 1.44	4.40 ± 2.52	3.70 ± 4.72	0.04	0.04
2.4% Propolis	8.48 ± 1.27	2.63 ± 1.61	3.70 ± 2.52	5.70 ± 1.15	0.04	0.06
3.6% Propolis	8.13 ± 1.81	0.90 ± 0.52*	1.70 ± 0.58	7.00 ± 5.00	0.02	0.07
DXR^b^	7.67 ± 1.87	5.97 ± 2.91	15.40 ± 4.16	15.00 ± 2.64	0.15	0.16

One-hundred metaphases were analyzed per culture, for a total of 300 cells per treatment.

^a^DMSO, dimethylsulfoxide, 0.5 *μ*L/mL, ^b^DXR, doxorubicin (1.0 and 2.0 *μ*g/mL in continuous and 3-h pulse treatment, resp.), ^c^Values are mean ± SD. *Significantly different from the control group (*P* < .05).

**Table 2:** Mean initial body weight, final body weight and body weight gain of rats and their respective control after 30 days of treatment with gels containing different concentrations of propolis.

Treatments (*n* = 5 rats/group)	Initial body weight (g)^a^	Final body weight (g)^a^	Body weight gain (g)^a^
Control	51 ± 6	322 ± 7	270 ± 6
Without propolis	47 ± 9	276 ± 11	229 ± 16
1.2% Propolis	48 ± 9	257 ± 25	209 ± 23
2.4% Propolis	51 ± 10	262 ± 38	211 ± 32
3.6% Propolis	50 ± 7	288 ± 49	239 ± 43

^a^Values are mean ± SD.

**Table 3:** Frequency of micronucleated polychromatic erythrocytes (MNPCEs) and nuclear division index (NDI) in peripheral blood of male Wistar rats submitted to acute, subacute and subchronic treatments with gels containing different concentrations of propolis and their respective controls.

Treatments (*n* = 5 rats/group)	Acute		Subacute		Subchronic	
	MNPCEs^a^	NDI^b^	MNPCEs^a^	NDI^b^	MNPCEs^a^	NDI^b^
Control	0.24	0.18 ± 0.06	0.09	0.14 ± 0.03	0.10	0.12 ± 0.02
Without propolis	0.32	0.21 ± 0.08	0.08	0.14 ± 0.04	0.03	0.10 ± 0.03
1.2% Propolis	0.06	0.20 ± 0.05	0.09	0.16 ± 0.03	0.06	0.13 ± 0.02
2.4% Propolis	0.28	0.16 ± 0.04	0.15	0.17 ± 0.05	0.05	0.12 ± 0.03
3.6% Propolis	0.28	0.20 ± 0.03	0.15	0.13 ± 0.02	0.03	0.12 ± 0.02
CPA^a^	0.89	0.15 ± 0.03	0.89	0.15 ± 0.03	0.89	0.15 ± 0.03

A total of 2000 cells were analyzed per animal, for a total of 10 000 cells per treatment.

^a^CPA, cyclophosphamide (50 mg/kg body weight), ^b^Values are percentage, ^c^Values are mean ± SD.
